# Effects of energetic ion irradiation on WSe_2_/SiC heterostructures

**DOI:** 10.1038/s41598-017-04042-8

**Published:** 2017-06-23

**Authors:** Tan Shi, Roger C. Walker, Igor Jovanovic, Joshua A. Robinson

**Affiliations:** 1 Department of Nuclear Engineering and Radiological Sciences, University of Michigan, Ann Arbor, MI 48109 USA; 2 Department of Materials Science and Engineering, The Pennsylvania State University, University Park, PA, 16802 USA; 3 Center for Two-Dimensional and Layered Materials, The Pennsylvania State University, University Park, PA, 16802 USA

## Abstract

The remarkable electronic properties of layered semiconducting transition metal dichalcogenides (TMDs) make them promising candidates for next-generation ultrathin, low-power, high-speed electronics. It has been suggested that electronics based upon ultra-thin TMDs may be appropriate for use in high radiation environments such as space. Here, we present the effects of irradiation by protons, iron, and silver ions at MeV-level energies on a WSe_2_/6H-SiC vertical heterostructure studied using XPS and UV-Vis-NIR spectroscopy. It was found that with 2 MeV protons, a fluence of 10^16^ protons/cm^2^ was necessary to induce a significant charge transfer from SiC to WSe_2_, where a reduction of valence band offset was observed. Simultaneously, a new absorption edge appeared at 1.1 eV below the conduction band of SiC. The irradiation with heavy ions at 10^16^ ions/cm^2^ converts WSe_2_ into a mixture of WO_x_ and Se-deficient WSe_2_. The valence band is also heavily altered due to oxidation and amorphization. However, these doses are in excess of the doses needed to damage TMD-based electronics due to defects generated in common dielectric and substrate materials. As such, the radiation stability of WSe_2_-based electronics is not expected to be limited by the radiation hardness of WSe_2_, but rather by the dielectric and substrate.

## Introduction

Layered transition metal dichalcogenides (TMDs) have received much attention in recent years because of their outstanding chemical^1^, optical^2^, and electronic properties. Tungsten diselenide (WSe_2_), a layered material with a direct optical bandgap of 1.65 eV in the monolayer form, exhibits attractive electronic properties such as high on/off current ratio^3^ and high mobility^4^, which make it a candidate for next-generation low-power electronic devices^5, 6^. Significant progress has been made in fabrication of WSe_2_ by controlled growth via metal-organic chemical vapor deposition^7^ and molecular beam epitaxy^8^. Novel transistors^9, 10^ and optoelectronic devices^11^ have been constructed based upon WSe_2_. However, the effects of ionizing radiation on WSe_2_ have not been extensively explored, and the understanding of radiation effects is critical for assessing the potential of WSe_2_-based electronics for use in high-radiation environments such as space. The principal component of cosmic rays are protons and heavy charged particles, and for this reason we have studied the damage induced by proton and heavy ion bombardment in WSe_2_/SiC heterostructures.

It is well known that ion bombardment can have a range of effects on other layered semiconducting materials^12^. Besides radiation damage, ion irradiation can also be used to tailor the material structure and properties. For example, low-energy ions can be used to introduce doping in a highly controlled fashion and thereby tune the electronic properties^13–15^. Focused ion beams can be used to precisely etch and pattern two-dimensional materials^16–18^. High-energy heavy ions usually have detrimental effects on material structure and device performance^19, 20^. The layered materials exhibit a unique response to ion irradiation in comparison to bulk materials due to their atomic-scale thickness, which can impact their sensitivity to environmental factors (such as oxygen and water)^21, 22^, as well as the mechanisms by which the energy is dissipated^23^. It has been shown that when graphene is irradiated with a proton beam, the defect generation is dependent on the number of graphene layers and is coupled to the interactions with the underlying substrate^24, 25^. Due to the reduced screening of electron-electron interactions in graphene, it has been proposed that, unlike conventional metals, graphene can be damaged through electronically stimulated desorption^25^. It has also been shown that the proton irradiation of MoS_2_ field effect transistors can induce trap states at interfaces and within the gate oxide, degrading the transistor’s performance^26^. Heavy ions can cause higher displacement damage than protons due to their higher atomic mass and energy transferred per collision. The effect of ion irradiation on structural defect and disorder generation^27^, electronic structure tuning^13, 28^, and electronic device performance degradation^29^ to WSe_2_ has been investigated in previously reported work.

Many previous irradiation studies of layered materials have focused on graphene and MoS_2_. Here we report on the chemical and optical modification and the electronic property changes to the heterostructure consisting of mechanically exfoliated WSe_2_ and bulk silicon carbide (6H-SiC) by proton and heavy ion irradiation using X-ray photoelectron spectroscopy (XPS) and UV-Vis-NIR spectroscopy. Although ions can produce damage along the entire length of their tracks, XPS, a surface characterization technique, was used for characterization of sample damage. This is because we are interested in the material modification near the WSe_2_ channel region, which would have a significant impact on the charge carrier transport in an electronic device based upon WSe_2_ material, such as a transistor. We investigate proton damage due to its significance for space applications, since cosmic radiation consists primarily of energetic protons. Although cosmic rays are mostly composed of protons and helium nuclei, it is also worth investigating the impact of heavier ions, which experience higher stopping power and are thus expected to produce higher damage rate. Heavy ions are a small but significant fraction of cosmic radiation. Furthermore, heavy ions can be used to simulate neutron radiation damage by virtue of their comparable damage mechanism and greatly reduced irradiation time^30–32^.

## Results

### Proton irradiation

Prior to proton irradiation, the stability of exfoliated WSe_2_ in vacuum and in air was evaluated. It is well-known that selenide-based TMDs can be oxidized in air at room temperature over long periods of time^33^. To determine if the oxidation is significant over the time frame of our experiment (several days), SiC with WSe_2_ surface flakes (WSe_2_/SiC) were prepared and characterized via XPS. Samples were then either left in air or stored inside a vacuum chamber under a medium vacuum (10–100 mTorr). After three days, the samples were removed and characterized again by XPS. Comparison of the spectra for the core tungsten shells (W 4 f; see Supplementary Fig. S1) reveals that only two visible peaks are observed in both cases, which are attributed to WSe_2_ (W 4 f_5/2_ and W 4 f_7/2_). For the sample in air, the binding energy of the W 4 f_7/2_ peak has an initial position of 32.31 eV that shifts upwards slightly to 32.40 eV. For the sample in vacuum, no significant shift was observed. These values for binding energy are in good agreement with the previously reported results for WSe_2_^3, 34^. Since oxidation is known to cause a downshift of the binding energy^34, 35^, it can be concluded that no significant oxidation of exfoliated WSe_2_ occurs over the studied time period for either case.

**Figure 1:**
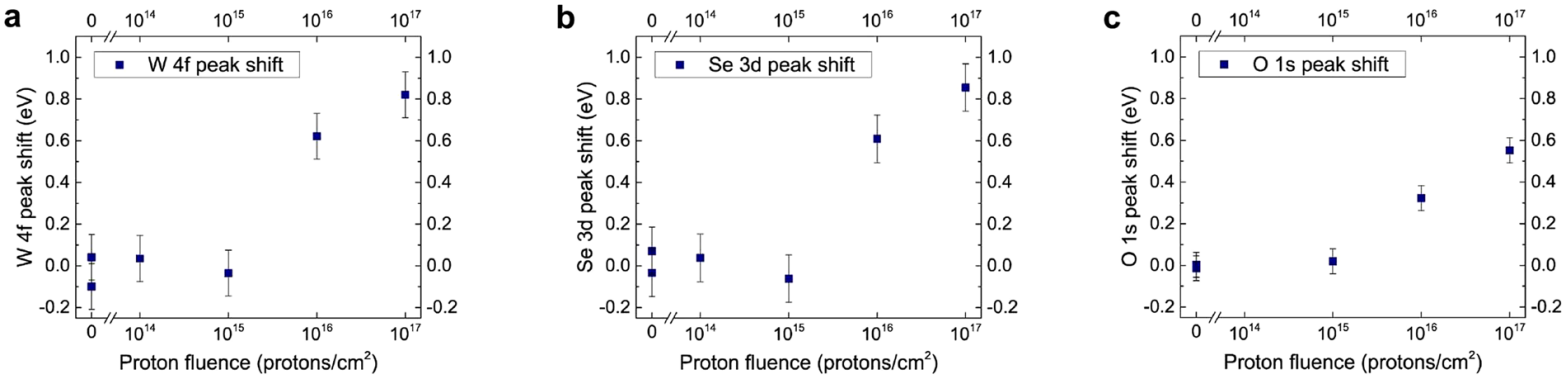
Binding energy shifts of WSe_2_/SiC heterostructure as a function of proton fluence. The shift of the peak position of (**a**) W 4 f_7/2_ (~32 eV) and (**b**) Se 3d_5/2_ (~54 eV) corresponding to the WSe_2_. (**c**) The binding energy shift of O 1 s peak (~532 eV) from surface oxygen. Data points at zero fluence correspond to control samples. A fluence at or above 10^16^ protons/cm^2^ is shown to induce a detectable shift in the XPS data.

After confirming that the samples do not oxidize in the experimental environmental conditions within the experimental time frame, samples of WSe_2_/SiC, bulk SiC, and bulk WSe_2_ were prepared and characterized by XPS. Samples were then either irradiated by 2 MeV protons or left unexposed as control samples. It is apparent from the XPS data that no oxidation (determined by the lack of appearance of tungsten oxide peaks as shown in Supplementary Fig. S2a.) was induced in the exfoliated WSe_2_ by proton beam irradiation. However, a variation in the position of W 4 f peaks has been observed that appears to depend on the proton fluence (Fig. 1a). No significant variation of the XPS spectra has been observed for the control samples or when the samples were irradiated at lower fluences (10^14^ and 10^15^ protons/cm^2^). However, a measurable increases in binding energy of ~0.6 eV and ~0.8 eV were observed for the samples irradiated at 10^16^ and 10^17^ protons/cm^2^, respectively. The same trend is observed for selenium (as measured via the Se 3d spectra; see Fig. 1b), and a similar trend was observed for oxygen (as measured via the O 1 s spectra; see Fig. 1c) where surface oxygen is attributed to the adsorption of oxygen onto the SiC substrate. However, the upshift for the O 1 s peak, ~0.3 eV at 10^16^ protons/cm^2^ and 0.5 eV at 10^17^ protons/cm^2^, is smaller than that of the WSe_2_ XPS signature. Significant shifts in peak positions for carbon (C 1 s) or silicon (Si 2p) were not observed until the 10^17^ protons/cm^2^ irradiation fluence was reached, and they were on the order of ~0.2 eV for C 1 s and ~0.3 eV for Si 2p. Since the XPS resolution is approximately 0.1 eV and there could be differential charging due to the nonuniformity of the exfoliated flakes, a shift of 0.2–0.3 eV for C 1 s and Si 2p peak might not be statistically significant. However, the W 4 f binding energy shift of 0.6 eV and 0.8 eV is significant when compared to the equipment resolution.

We attribute the observed peak shifts to charge buildup resulting from proton/substrate interactions. The 2 MeV protons penetrate 32 μm deep into the SiC substrate, as estimated by the SRIM/TRIM software package^36^. A proton loses energy along its track via two mechanisms: inelastic collisions with bound electrons in the medium (electronic ionization and excitation) and elastic collisions with nuclei. The electronic stopping dominates at short depths, where the layered WSe_2_ is located, while nuclear stopping only dominates at the end of its range (see Supplementary Fig. S3 for the depth profile of proton-induced ionization and dpa in 6H-SiC). Since a proton is relatively light in comparison to the nuclei constituting the irradiated sample, the probability of displacement damage to the surface material is very small (~0.007 dpa for a fluence of 10^17^ protons/cm^2^ in the first 10 nm of the WSe_2_ surface, as calculated by SRIM/TRIM). This is consistent with the lack of oxidation peak, as measured by XPS after the irradiation. The ionization is responsible for the charge transfer at the WSe_2_/SiC interface, which causes the XPS core-level peak shift. Although a small positive shift of W 4 f peak to the order of ~0.15 eV can be induced by extensive oxidation^37, 38^ and there could be oxidation below the detection limit of XPS, the relatively large W 4 f peak shift (~0.6 eV for a fluence of 10^16^ protons/cm^2^ and ~0.8 eV for a fluence of 10^17^ protons/cm^2^) observed in this work should be mostly attributed to the charge transfer at the WSe_2_ interface. Based on SRIM/TRIM simulations, the electronic energy loss within the WSe_2_ and SiC at the sample surface is approximately 56 eV/nm and 39 eV/nm, respectively. Since the probability of direct interaction between a proton and WSe_2_ is small, most of the interactions occur within the substrate. The onset of a measurable charging effect in the WSe_2_ is seen at the fluence of 10^16^ protons/cm^2^. This fluence corresponds to a relatively high radiation dose in comparison to doses known to induce effects on the operation of TMD transistors. For example, trapped charges in dielectrics such as silicon dioxide can degrade the device electrical characteristics of TMD transistors at a dose level that is two to three orders of magnitude lower than used in this experiment^26^, suggesting that degradation in TMD-based device architectures is not due to the TMD layer, but surrounding materials. The XPS peak shift observed in our experiment can be interpreted as charge transfer due to a combination of direct damage to WSe_2_, indirect effects from the substrate, and carrier trapping by interface states.

Samples of bulk WSe_2_ and SiC were also exposed to protons at 10^16^ protons/cm^2^ in order to analyze the effect of proton damage on the band alignment between these two semiconductors. To the best of our knowledge, this is the first attempt to measure the valence band offset (VBO) between these two materials using XPS. The VBO between these materials was determined from the following equation^39–41^.

1 $${\rm{\Delta }}{E}_{v}={\rm{\Delta }}{E}_{CL}(i)+({E}_{W\mathrm{\ 4}f}^{WS{e}_{2}}-{E}_{VBM}^{WS{e}_{2}})+({E}_{Si\mathrm{\ 2}p}^{6H \mbox{-} SiC}-{E}_{VBM}^{6H \mbox{-} SiC}),$$

**Figure 2:**
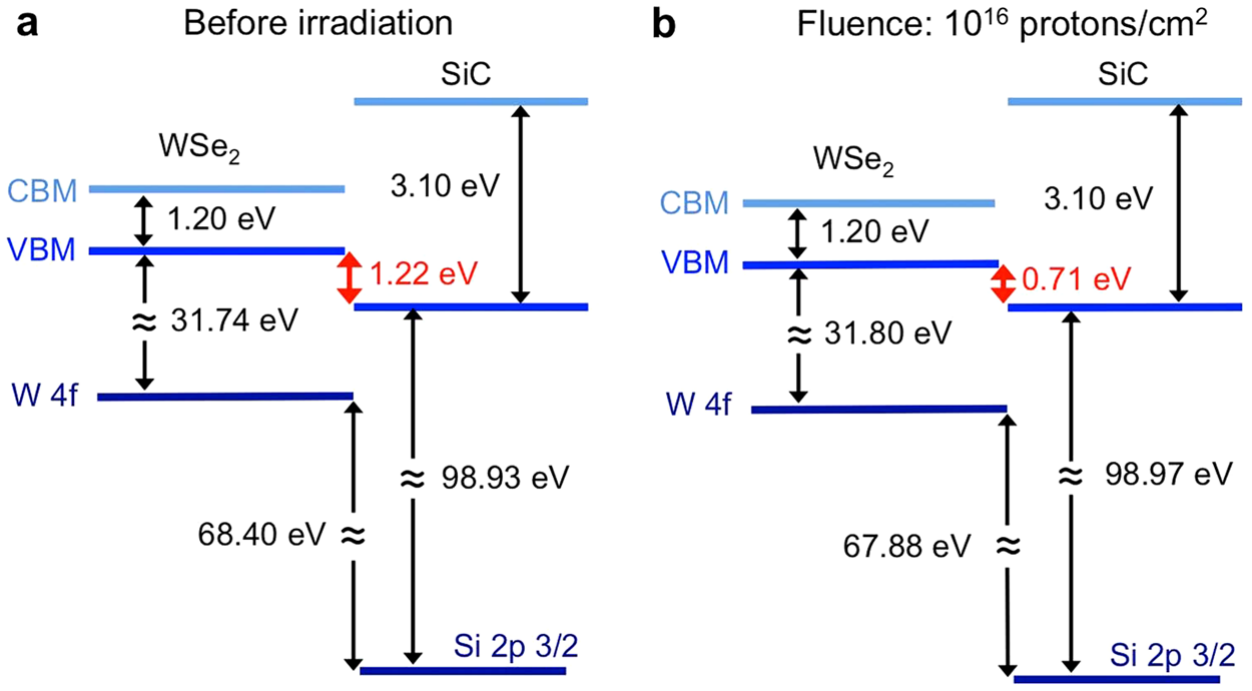
Valence band offset between WSe_2_ and SiC in the WSe_2_/SiC heterostructure. The VBO was calculated using XPS spectra and was found to decrease after proton irradiation. It is assumed that the bandgap was not modified by the proton beam exposure (CBM: conduction band minimum).

Here, Δ*E*_*CL*_(*i*) is the energy difference between the two selected core shell states of the studied heterojunction (in this case, between W 4 f and Si 2p states of the WSe_2_/SiC heterostructure). The band alignment before and after radiation damage is schematically shown in Fig. 2a and b. The core level separation at the interface is determined to have an initial value of 68.4 eV, decreasing to 67.9 eV upon proton beam exposure. This decrease in the core level separation originates from the charging processes described above. Interface states would not affect the VBO because they would contribute to the WSe_2_ and SiC equally and their contribution to VBO would cancel out^39^. Valence band maximum (VBM) was measured by applying a linear regression to the low binding energy edge of the valence band spectrum to both the bulk WSe_2_ and the bulk SiC samples. As shown in Fig. 2, the VBO between WSe_2_ and SiC decreases from 1.22 ± 0.24 eV to 0.71 ± 0.24 eV after proton beam exposure at 10^16^ protons/cm^2^. Spectra used for the calculation of VBO are shown in Supplementary Fig. S2. The error bar of 0.24 eV is estimated from the error of six measurement values, where an error of 0.1 eV for each measurement is used. The proton beam exposure had a minimal effect on the separation between the core-level peak and VBM of bulk SiC or bulk WSe_2_. The primary cause of the VBO change is the shift of the W 4 f peak relative to the Si 2p peak in the WSe_2_/SiC heterostructure.

**Figure 3:**
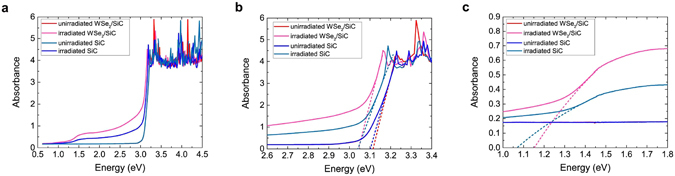
UV-Vis-NIR spectra before and after 2 MeV proton irradiation at a fluence of 10^16^ protons/cm^2^. (**a**) The measured UV-Vis-NIR spectra reveal irradiation-induced changes in the optical properties of the SiC substrate. Magnified regions of the plot (**a**) depicting the changes in the absorption edge corresponding to (**b**) the SiC band gap and (**c**) the new absorption edge created around 1.1 eV due to the irradiation-induced vacancy-rich region deep in the SiC substrate.

Regarding the ion irradiation effects on the SiC substrate, the dependence of amorphization and annealing on dose and temperature, as well as other property modifications, have been previously studied^42, 43^. The conversion of the crystalline and transparent SiC to a heavily defective and either darkened (partially translucent) or black (opaque) Si_*x*_C_*y*_ material was observed deep within the substrate for the samples exposed to 10^16^ and 10^17^ protons/cm^2^, respectively (see Supplementary Fig. S4). The color change is also witnessed in a sample of exfoliated MoS_2_ on SiC substrate after exposure to 2 MeV 10^16^ protons/cm^2^. In light of this change, the light absorbance properties of a control sample and sample exposed to 10^16^ protons/cm^2^ was measured in order to examine how the color change in proton-irradiated SiC correlates to optical properties. While XPS reveals the information on the chemical bondings within the first ~10 nm of the surface, the UV-Vis-NIR spectroscopy provides an absorption spectrum of the entire sample in the lower energy range of the electromagnetic spectrum. The absorbance data obtained are shown in Fig. 3. For all samples, there is a sharp increase of light absorbance for photons with energy at or above 3 eV, corresponding to the band gap of 6H-SiC (Fig. 3b). Additionally, a new feature appears in the irradiated samples at ~1.5 eV, with an absorption edge at ~1.1 eV (Fig. 3c). The absorbance of light due to this feature appears to result in the black color of the irradiated SiC, as this energy lies in the near-infrared part of the spectrum. This is explained by the generation of a deep acceptor in the SiC band gap near E_*c*_-1.1 eV^44, 45^. This deep acceptor results from point defects such as the silicon single vacancy, the carbon single vacancy, and carbon anti-sites^46, 47^.

### Heavy ion irradiation

When a heavy ion interacts with matter, although electronic energy transfer creates excitations and displacements in the target lattice, collision cascades induced by the nuclear stopping power are the dominant damage mechanism in semiconducting and metallic materials. Table 1 shows the results of SRIM/TRIM simulations of the three irradiation conditions investigated in this work. Bulk material parameters for WSe_2_ were used in the simulation since exfoliated WSe_2_ has a relatively large average thickness (see Supplementary Fig. S5). The displacement threshold energy was set to 25 eV for W and Se for the calculation of dpa. For monolayer WSe_2_, the displacement threshold energy is calculated to be ~6.4 eV based on DFT molecular dynamics simulation, assuming a 5 × 5 supercell of WSe_2_ monolayer^48^. Fewer layers correspond to a lower displacement threshold, which results in a higher damage rate^25^. The coupling with the substrate would also affect the displacement threshold^25^. The penetration depth of the heavy ions used in the experiment is in the range of 1–2 μm. The nuclear stopping power of 2.5 MeV Fe ions is higher than that of 5 MeV Fe ions at the sample surface while the opposite is true for electronic stopping power. The dpa of 4 MeV Ag ions is approximately 2–3 times higher than that of Fe ions and is therefore expected to result in a higher structural damage if the damage is not saturated.

**Table 1:** Summary of the results from SRIM/TRIM simulation.

	Projected range in bulk WSe_2_ (μm)	(*dE*/*dx*)_*nucl*_ at WSe_2_ surface (MeV cm^2^/mg)	(*dE*/*dx*)_*elec*._ at WSe_2_ surface (MeV cm^2^/mg)	*Dpa* in WSe_2_
2.5 MeV Fe^2+^ ion	1.00	0.47	2.04	3.7
5 MeV Fe^4+^ ion	1.73	0.30	3.58	2.3
4 MeV Ag^4+^ ion	0.93	1.51	1.98	8.7
	**Projected range in bulk SiC (μm)**	**(** ***dE*** **/** ***dx*** **)**_***nucl***_ **at SiC surface (MeV cm** ^**2**^ **/mg)**	**(** ***dE*** **/** ***dx*** **)**_***elec***_. **at SiC surface (MeV cm** ^**2**^ **/mg)**	***Dpa*** **in SiC**
2.5 MeV Fe^2+^ ion	1.24	0.90	7.16	0.74
5 MeV Fe^4+^ ion	1.98	0.55	12.1	0.47
4 MeV Ag^4+^ ion	1.23	6.99	2.76	1.6

The dpa is calculated with a fluence of 10^16^ ions/cm^2^ at the top 10 nm of the material, corresponding to the detection depth of XPS measurement. The monolayer collisions calculation type was used in the SRIM/TRIM simulation. The depth profiles of ionization and dpa in the SiC substrate induced by heavy ions are shown in Supplementary Fig. S3.

**Figure 4:**
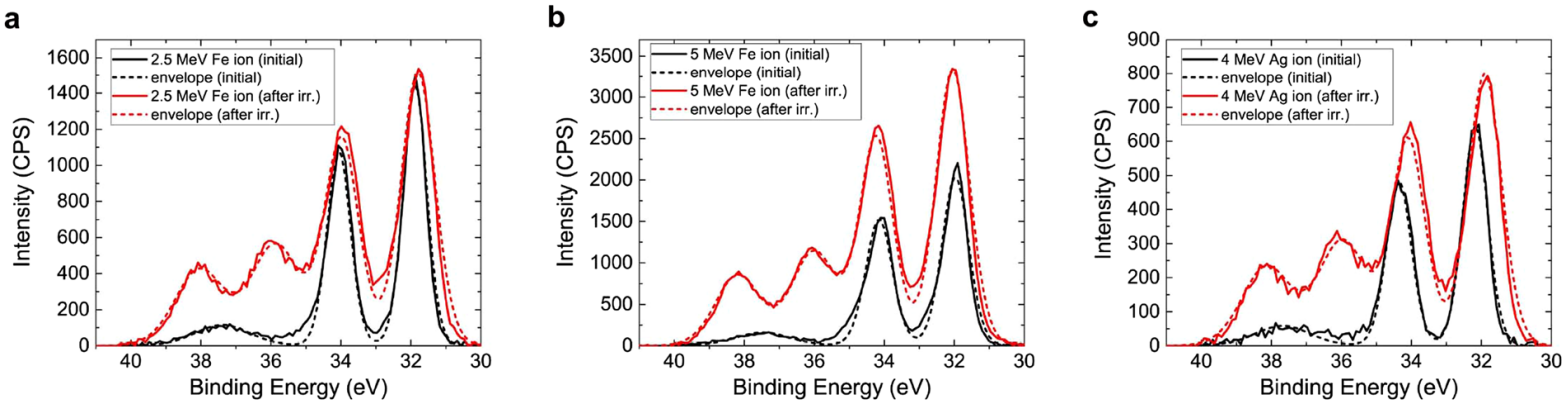
Change of XPS W 4 f peak spectra after heavy ion irradiation. The change of XPS W 4 f spectrum after irradiation with (**a**) 2.5 MeV Fe, (**b**) 5 MeV Fe, and (**c**) 4 MeV Ag ions at a fluence level of 10^16^ ions/cm^2^. The XPS spectrum intensity is expressed in the units of counts per second (CPS). The appearance of two additional peaks at ~36 eV and ~38 eV after irradiation indicates the oxidation of tungsten.

**Figure 5:**
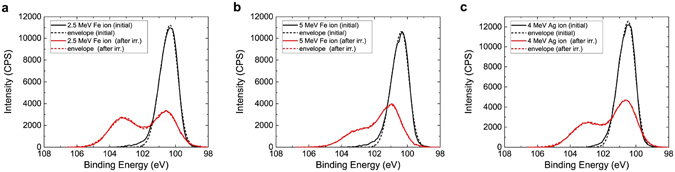
Change of XPS Si 2p_3/2_ spectra after heavy ion irradiation. The change of XPS Si 2p_3/2_ spectrum after irradiation with (**a**) 5 MeV Fe, (**b**) 2.5 MeV Fe, and (**c**) 4 MeV Ag ions at a fluence of 10^16^ ions/cm^2^. The appearance of new peak indicates the oxidation of silicon.

**Figure 6:**
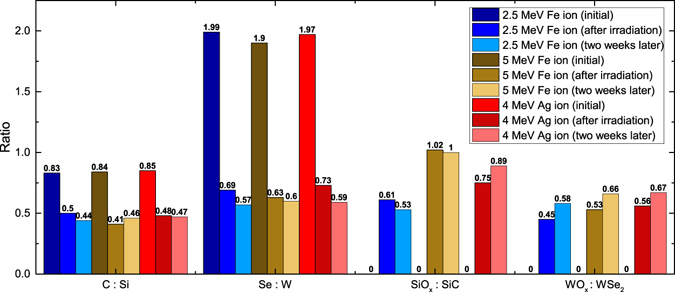
Compositional analysis of WSe_2_/SiC heterostructure from XPS studies. The compositional ratio of C:Si, Se:W, SiO_*x*_:SiC and WO_*x*_:WSe_2_ of WSe_2_/SiC heterostructure before and after heavy ion irradiation. No oxidation of silicon and tungsten has been detected before irradiation.

While MeV-level proton beam exposure leads to charging effects in the WSe_2_ without significant chemical modification, exposure to heavy ion beams at MeV energies leads to a partial transformation of WSe_2_ into tungsten oxide (see Fig. 4), as well as a partial transformation of SiC into SiO_2_ and heavily defective SiC mixture (see Fig. 5). Compared to protons, the probability of elastic collisions and the average energy transferred to the primary knock-on atom were much higher for heavy ions, which led to larger, higher-density collision cascades. Due to the higher nuclear stopping power as reflected by the dpa values, the effects of sputtering, recoil mixing, and cascade mixing were more pronounced^30^, which caused significant structural damage and generated a significant density of dangling bonds at the surface. The ion beam exposure was carried out under high vacuum conditions (<10^−8^ Torr), and therefore the oxidation occurs due to the exposure to atmosphere during *ex-situ* XPS analysis. Initial XPS reveals that tungsten and selenium are only bonded to each other, corresponding to WSe_2_. After heavy ion irradiation, a second set of peaks emerges in the tungsten spectra that we assign to tungsten oxide based on peak positions of ~36.0 eV and ~38.2 eV^34^ and the changes in the oxygen spectra that appear to indicate the formation of a metal oxide (see Supplementary Fig. S6). At the same time, there is a drastic reduction in the amount of selenium relative to the amount of tungsten. The initial Se:W ratio for these three samples is 1.96 ± 0.05 and is reduced to 0.68 ± 0.05 following heavy ion bombardment (Fig. 6). This can be explained by the higher volatility of selenium compared to tungsten. We estimate using XPS that the ratio of tungsten-oxygen bonding to tungsten-selenium bonding is 0.51 ± 0.05 after the beam exposure, and increases slightly after two weeks in storage due to continued oxidation. The Se:W ratio was also observed to decrease from a measurement made two weeks following the experiment, confirming the continued desorption of selenium from the sample. Although the dpa value of 4 MeV Ag is the highest among the three experiments and the dpa of 2.5 MeV Fe is the lowest, the magnitude of damage estimated on the basis of Se:W and WO_*x*_:WSe_2_ ratio is very similar in all three experiments and does not exhibit a clear trend. At the total ionizing dose levels used in this experiment, the amount of damage caused by the three ions to the WSe_2_ may be similar enough that the different samples oxidize by roughly the same amount when exposed to air. Comparisons between samples exposed to varying dose levels will be needed to confirm if a correlation between dpa and oxidation exists. This correlation was studied for several other materials^49, 50^ and the same methodology could be applied to study the oxidation of layered materials. Other characterization techniques such Rutherford backscattering spectrometry and cross-sectional transmission electron microscopy could be used to study the induced damage at greater depths in the substrate.

A change of composition is also observed for SiC, where the C:Si ratio drops from 0.84 ± 0.01 to 0.47 ± 0.025 (Fig. 6) post irradiation. The initial spectrum for silicon reveals only one chemical state: a peak doublet corresponding to silicon carbide. After the irradiation, another doublet centered around ~103 eV was created that we attribute to a silicon-oxygen compound^51^, with silicon in a Si^2+^ or Si^3+^ state (Fig. 5). Carbon is initially present in three states: carbon-silicon bonds; carbon-carbon sp^3^ bonds; and carbon-oxygen bonds. Carbon-carbon and carbon-oxygen bonds are both attributed to adventitious surface carbon. These same states were observed after irradiation, but in different proportions—more of the carbon signal can be attributed to the two surface carbon states rather than to the SiC. Additionally, the binding energy of carbon determined from the carbon peak corresponding to SiC increases from 282.6 eV to 283.4 eV. The carbon binding energy for SiC in the range between 282.9 eV and 283.5 eV has been previously reported for SiC whiskers^52^ and amorphous SiC^53^. We could conclude that this shift corresponds to a transformation of the SiC from crystalline to amorphous. Complete amorphization of 6H-SiC corresponds to a damage level of ~0.5–10 dpa, depending on the ion energy and species^43^, and is thus expected.

**Figure 7:**
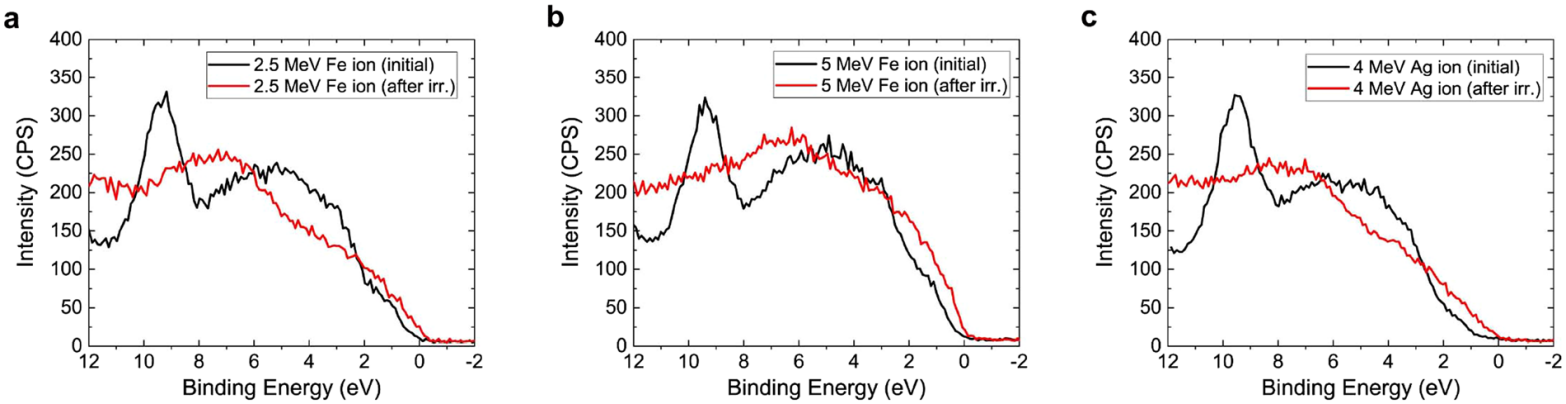
Change of valence band spectra after heavy ion irradiation. The change of XPS spectrum near the valence band region after irradiation with (**a**) 2.5 MeV Fe, (**b**) 5 MeV Fe, and (**c**) 4 MeV Ag ions at a fluence of 10^16^ ions/cm^2^.

A significant modification of the valence band structure was also observed via XPS (Fig. 7). The initial spectrum consists of a single peak that is attributed to the SiC, as well as a broader feature at a lower energy that is a convolution of the SiC and WSe_2_ valence orbitals. The VBM of the semi-insulating SiC substrate is typically located at ~1.5 eV. Measurement of the VBM using a linear fit to the valence band edge reveals an average value of 0.36 eV due to the initial p-type doping of the exfoliated WSe_2_. After irradiation, all samples show a shape change in the valence band spectra and lead to a VBM at or below the Fermi level using the linear fit approach, which indicates a loss of the semiconducting properties of the heterostructure. Additionally, the single peak has also vanished from the spectra due to heavy ion damage. The increases in the signal intensity around 8 eV and 12 eV are attributed to tungsten oxide^54^ and silicon oxide^55^ contributions to the valence band.

## Conclusions

The effects of high energy proton and heavy ion bombardment on the WSe_2_ and SiC heterostructure have been studied with XPS and UV-Vis-NIR spectroscopy. We have found that a proton fluence of ~10^16^ protons/cm^2^ is needed to influence the surface chemistry, band offset, and absorbance properties. No new surface states are generated by proton exposure, but a charging effect is observed that is mostly attributed to the proton-induced ionization and excitation within both the WSe_2_ and the SiC substrate. The difference in charging between WSe_2_ and SiC leads to a change in the valence band offset, as measured by XPS. The buildup of point vacancies, such as the silicon single vacancy, carbon single vacancy, and carbon anti-site also result in the modification of light absorbance properties within the SiC substrate. These two different effects would both have consequences for device performance based on this heterostructure, which should be evaluated in future work. The bombardment of WSe_2_/SiC heterostructure with heavy ions at high dpa levels leads to significant physical damage. Collisions between heavy ions and WSe_2_ lead to structural disorder and the preferential ejection of selenium; therefore, the sample is oxidized once exposed to an oxygen-bearing ambient. Collisions between heavy ions and SiC lead to the sputtering of both elements, but carbon is preferentially removed. These combined changes lead to heavy alteration of the band structure, which can be monitored by changes in the valence band spectra in XPS. We thus expect that at this total ionizing radiation dose level, a device based on this heterostructure would be damaged beyond repair due to heavy damage to the WSe_2_ channel. In conclusion, due to the absence of chemical modifications of WSe_2_ via XPS and the observation of charging effect only at very high proton fluence level, we expect the radiation resilience of TMD-based transistors to be limited by the dielectric insulator and the substrate, which would degrade at a much lower proton fluence level^26, 56^. The WSe_2_ structural change after heavy ion bombardment indicates the impact of nuclear stopping power on these ultra-thin materials. Further experiments to determine the threshold dose level to heavy ions, the dependence of ion-induced damage on the number of WSe_2_ layers and growth techniques, as well as experiments tracking damage to devices such as diodes and transistors, would aid to develop a more complete understanding of the radiation hardness of WSe_2_/SiC heterostructures and help to develop radiation-hardened systems based upon those materials.

## Methods

Three types of samples were prepared: SiC with WSe_2_ surface flakes, SiC with no WSe_2_ surface flakes, and mm-scale bulk WSe_2_ crystals. 400 μm thick semi-insulating 6H-SiC wafers were obtained from II-VI Advanced Materials, while bulk WSe_2_ crystals were obtained from 2Dsemiconductors, Inc. WSe_2_/SiC heterostructures were prepared by mechanically exfoliating WSe_2_ flakes from a bulk crystal and depositing them onto a sample of 6H-SiC (0001). The thickness of the flakes ranges from several layers to several microns (see Supplementary Fig. S5).

Proton and heavy ion irradiation was carried out at the Michigan Ion Beam Laboratory (University of Michigan). Irradiation with 2 MeV protons was performed at several fluences (10^14^, 10^15^, 10^16^, and 10^17^ protons/cm^2^). The proton fluence experienced by space electronics during their typical lifetime is within the range of fluences investigated experimentally in this work^57^. The heavy ion experiments were performed with 2.5 MeV Fe ions, 5 MeV Fe ions, and 4 MeV Ag ions at a fluence level of 10^16^ ions/cm^2^. The ion beam was raster-scanned over the sample area of 6 × 6 mm^2^ at an angle of ~7 degrees from the normal to sample surface to avoid channeling effects. The ion beam current density was kept within the range of 300–500 nA. The irradiation temperature was monitored in real time by thermal imager and kept between 50 degrees C and 100 degrees C to avoid selenium desorption due to thermal effects. The samples were stored in a vacuum chamber before and after the irradiation at a typical pressure of 0.1 Torr. The XPS was performed one day before irradiation, two to three days after irradiation, and two weeks after irradiation. XPS measurements to determine the stability of the samples in air and medium-level vacuum were performed using a Kratos Axis Ultra spectrometer, while the XPS measurements for irradiated samples were performed using a PHI VersaProbe II spectrometer. Both XPS tools use aluminum K_*α*_ X-rays (1486.6 eV). All samples were charge-referenced to adventitious carbon at 284.8 eV and mixed Gaussian-Lorentzian peak fits were used. Low energy electron flood gun was used during the XPS measurement for charge compensation. The UV-Vis-NIR spectroscopy was performed using a Perkin-Elmer Lambda 950 spectrophotometer. Transmittance data was collected for wavelengths ranging from 250 nm to 2000 nm, and then converted into absorbance as a function of photon energy.

## Electronic supplementary material

Supplementary material
