# Supplementary material for: Effects of energetic ion irradiation on WSe2/SiC heterostructures
Source: Sci Rep. 2017 Jun 23;7:4151. doi: 10.1038/s41598-017-04042-8 (PMC5482891; doi:10.1038/s41598-017-04042-8)
Supplement: Supplementary file 1 — Supplementary material [file 41598_2017_4042_MOESM1_ESM.pdf]

Supplementary Material for “Effects of energetic ion irradiation on WSe_2/SiC heterostructures”

Tan Shi[1,+], Roger C. Walker II[2,3,+], Igor Jovanovic[1,*], and Joshua A. Robinson[2,3,*]

[1]Department of Nuclear Engineering and Radiological Sciences, University of Michigan, Ann Arbor, MI 48109, USA
[2]Department of Materials Science and Engineering, The Pennsylvania State University, University Park, PA 16802, USA
[3]Center for Two-Dimensional and Layered Materials, The Pennsylvania State University, University Park, PA 16802, USA
[*]Corresponding author: ijov@umich.edu, jrobinson@psu.edu
[+]Both authors contributed equally to this work

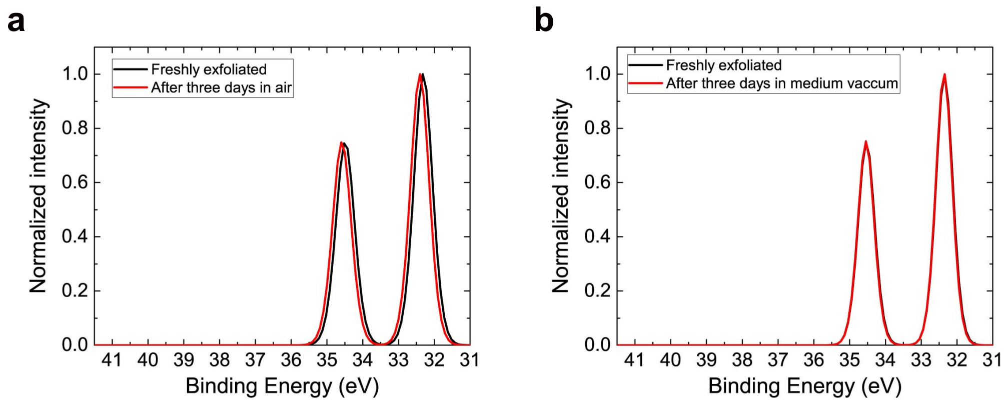

Figure S1. Stability of exfoliated WSe_2. The stability of the exfoliated flakes in (a) medium vacuum and (b) air has been demonstrated via XPS. The two peaks in the W $4f_{7/2}$ window both correspond to WSe_2. No significant tungsten oxide formation was found to occur within the time frame of these measurements. The initial W $4f_{7/2}$ positions and shifts were (a) 32.35 eV, + 0.01 eV; (b) 32.31 eV, + 0.09 eV.

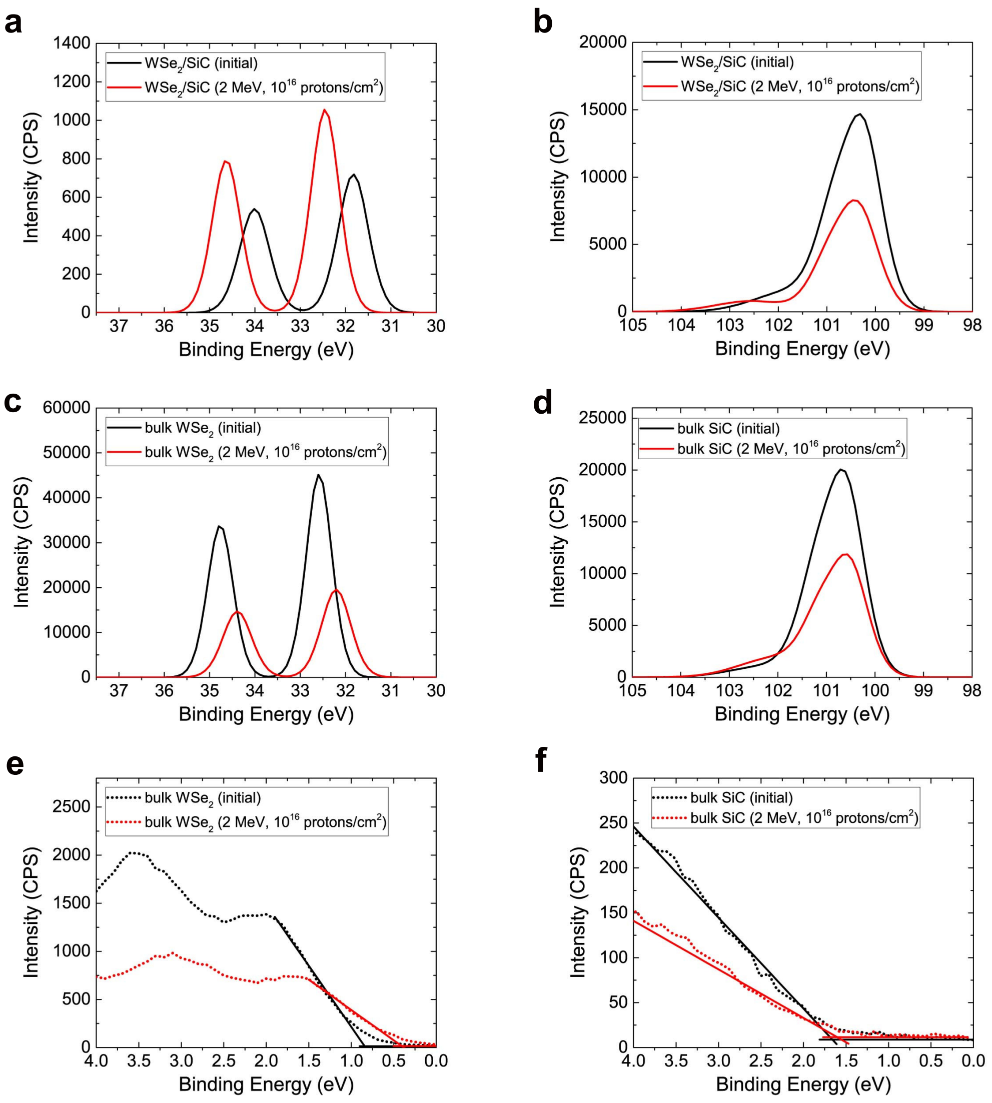

Figure S2. XPS spectra used in the calculation of valence band offset (VBO) between WSe_2 and SiC upon exposure with 2-MeV protons at a fluence of 10^{16} protons/cm^2. The VBO between WSe_2 and SiC in the WSe_2/SiC heterostructure can be calculated using XPS by determining the binding energy difference between (a) W 4f and (b) Si 2p in WSe_2/SiC heterostructure, energy difference between (c) W 4f and (e) valence band edge in bulk WSe_2, and energy difference between (d) Si 2p and (f) valence band edge in bulk SiC.

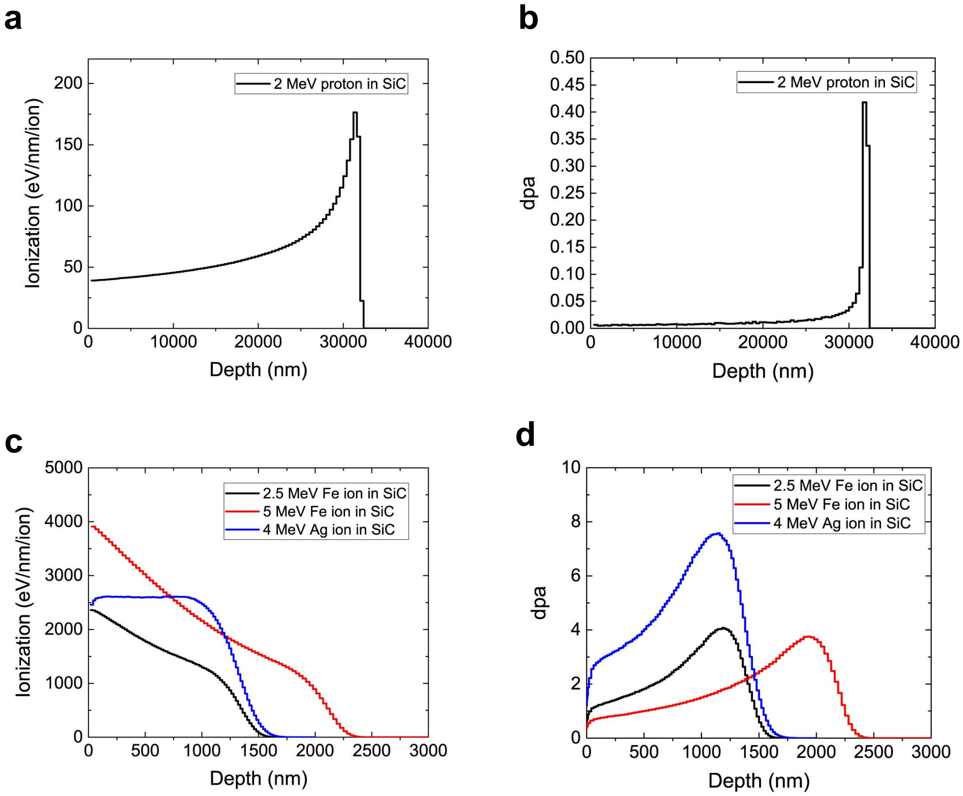

Figure S3. **The depth profile of ionization and dpa in SiC estimated from SRIM/TRIM simulation** (a) Ionization depth profile of 2 MeV proton in SiC; (b) dpa of 2 MeV proton in SiC at a fluence of 10^{17} protons/cm^2; (c) ionization depth profile of heavy ions in SiC; (d) dpa of heavy ions in SiC at a fluence of 10^{16} protons/cm^2. We used the 6H-SiC displacement threshold energies recommended by Debelle *et al.* (20 eV for C and 35 eV for Si)[1]. The monolayer collisions calculation type was used in the SRIM/TRIM simulation.

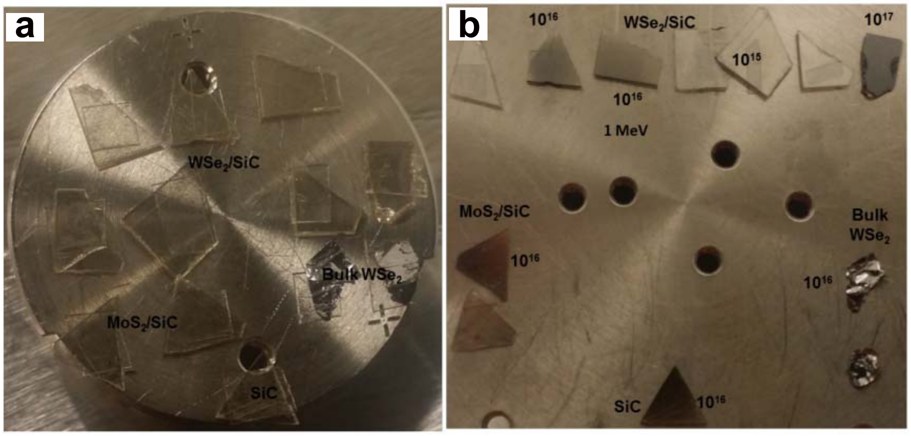

Figure S4. **Proton-irradiated samples before and after irradiation.** (a) Digital camera image of the TMD samples before irradiation. The samples are transparent due to the wide band gap of SiC and the nanoscale dimensions of the WSe_2 and MoS_2. (b) Digital camera image of the TMD samples after proton irradiation. Control samples (unlabeled) and samples exposed to 10^{15} protons/cm^2 are mostly transparent. Samples exposed to 10^{16} protons/cm^2 and 10^{17} protons/cm^2 turn black due to proton beam damage.

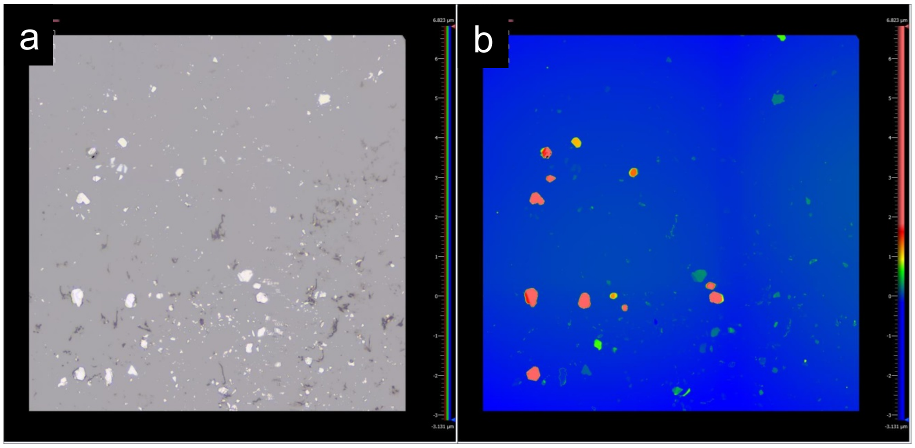

Figure S5. Optical image of exfoliated WSe_2. (a) Optical image of exfoliated WSe_2 flakes on 6H-SiC substrate taken by white light interferometry (optical profilometry). (b) Using this technique, the thickness of the WSe_2 flakes can also be measured. The thickness ranges from several layers to several microns. The sparse coverage of exfoliated material allows the substrate to be examined in XPS measurements.

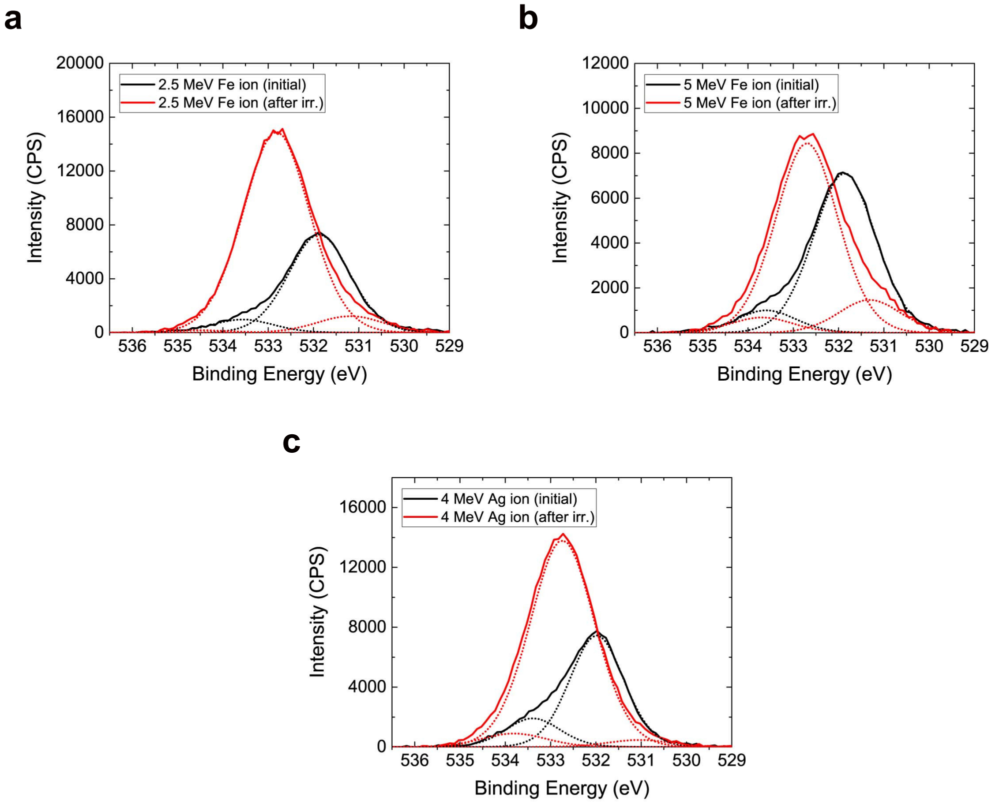

Figure S6. Change of O 1s peak spectrum after heavy ion irradiation. Oxygen 1s spectra before (black) and after the irradiation (red) for sample exposed to the heavy ions. The initial oxygen spectra contain two chemical environments – one for adsorbed moisture and the other for organic contaminants. The final spectra contain those two peaks and a third corresponding to metal oxides.

References

1. Debelle, A. *et al.* Characterization and modelling of the ion-irradiation induced disorder in 6H-SiC and 3C-SiC single crystals. *Journal of Physics D: Applied Physics* **43**, 455408 (2010).
